# Supplementary material for: Comprehensive Annotation and Functional Exploration of MicroRNAs in Lettuce
Source: Front Plant Sci. 2021 Dec 24;12:781836. doi: 10.3389/fpls.2021.781836 (PMC8739914; doi:10.3389/fpls.2021.781836)
Supplement: Supplementary file 3 [file Data_Sheet_9.ZIP › GSM2230751.plot/Lsa-miR393c_Lsat_1_v5_gn_4_31720.1_818_TPlot.pdf]

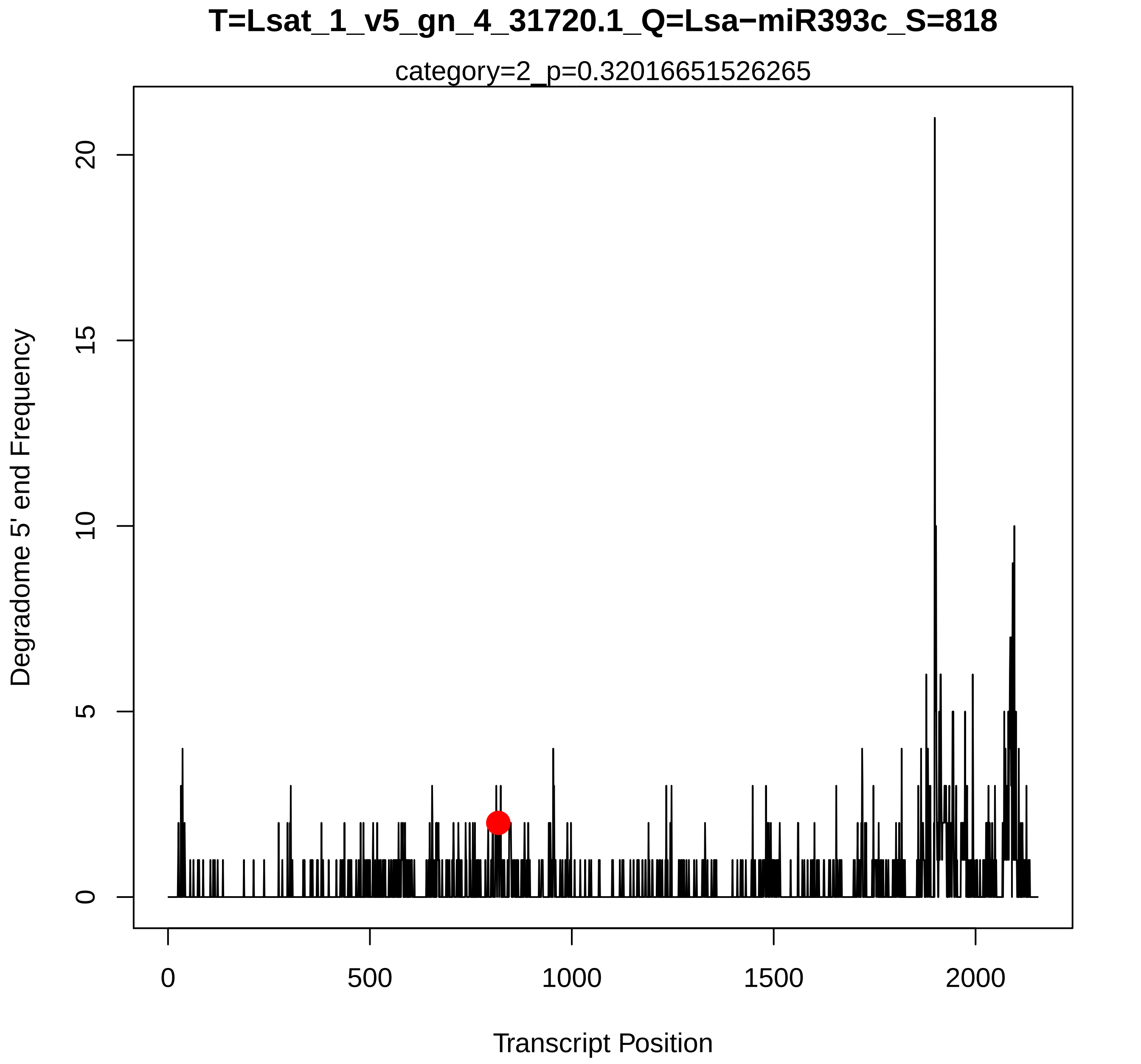

T=Lsat_1_v5_gn_4_31720.1_Q=Lsa−miR393c_S=818
category=2_p=0.32016651526265
Degradome 5' end Frequency
0
5
10
15
20
0
500
1000
1500
2000
Transcript Position
